# Interactions between sleep habits and self-control

**DOI:** 10.3389/fnhum.2015.00284

**Published:** 2015-05-11

**Authors:** June J. Pilcher, Drew M. Morris, Janet Donnelly, Hayley B. Feigl

**Affiliations:** Department of Psychology, Clemson UniversityClemson, SC, USA

**Keywords:** sleep, self-control, glucose, motivation, effort, physiological resources, prefrontal cortex, controlled attention model

## Abstract

Good sleep habits and effective self-control are important components of successful functioning. Unfortunately chronic sleep loss and impaired self-control are common occurrences for many individuals which can lead to difficulty with daily self-control issues such as resisting impulses and maintaining attentive behavior. Understanding how self-control is depleted and how good sleep habits may help replenish and maintain the capacity for self-control is an important issue. A sleep-deprived individual who has expended the necessary resources for self-control is at an increased risk for succumbing to impulsive desires, poor attentional capacity, and compromised decision making. To date, few studies have investigated how sleep and self-control are inter-related. The goal of this mini-review is to explore the intersection between sleep habits and self-control and encourage researchers to focus on a new area of research that integrates what are at present largely separate areas in psychology and human neurosciences.

Twenty-four-hour-a-day and world-wide operations create an environment where workers sleep less or sleep at irregular times often resulting in poor sleep and chronic sleep loss for many adults (Bonnet and Arand, 1995). Although many studies have examined the effects of sleep deprivation (e.g., Lim and Dinges, 2010), fewer studies have focused on the effects of poor sleep habits on functioning. Furthermore, there have been limited attempts to develop a theoretical approach that could provide a means of better understanding and predicting the effects of poor sleep habits and sleep deprivation. One recent approach to understanding how sleep deprivation impacts performance is the Controlled Attention Model (Pilcher et al., 2007). The Controlled Attention Model maintains that sleep-deprived individuals will perform worse on tasks that require effortful attention to complete. As such, the model focuses on the basic human capability of monitoring and directing attention; indicating that sleep deprivation could negatively impact self-control. The purpose of this paper is to explore how sleep habits and self-control are interwoven and how sleep habits and self-control may work together to affect daily functioning.

Self-control is an important part of daily decision making. It provides the opportunity to monitor and alter one’s responses. It allows one to maintain control when presented with conflicting desires and opportunities (Baumeister and Vohs, 2007). Self-control often includes resisting pleasurable impulses to better meet long-term goals (Hofmann et al., 2009). The ability to choose goals and monitor progress toward the goals as well as the ability to regulate social behaviors are also part of self-control (Baumeister et al., 2007). As such, self-control contributes to the ability to be flexible and adapt to new environmental and social demands. The capacity for self-control; however, is limited in many situations. For example, making repeated choices makes it harder for many individuals to continue to exert self-control. Common circumstances that lead to problems with self-control include experiencing negative moods, allowing small mistakes to lead to bigger mistakes, and trying to resist multiple temptations (Heatherton and Wagner, 2011).

Another common condition that could negatively impact self-control is poor sleep habits. Poor sleep habits include inconsistent sleep times and inadequate sleep quantity (Barber et al., 2010). As such, poor sleep habits comprise sleep-related events such as low sleep efficiency (i.e., lying awake in bed when trying to sleep), poor sleep quality, and sleep deprivation. Poor sleep is associated with sensitive periods of micro-arousals (Pilcher and Schulz, 1987) and can result in on-the-job sleepiness (Mullins et al., 2014). Poor sleep and sleep deprivation are risk factors for health-related incidents such as weight gain, hypertension, and illness (Patel and Hu, 2008; Cohen et al., 2009; McCubbin et al., 2010). More specifically, sleep deprivation has a negative impact on metabolism and endocrine function (Spiegel et al., 1999) as well as a negative impact on responsivity to stimuli resulting in reduced attention (Boonstra et al., 2007). Furthermore, Walker et al. (2009) suggest that sleep deprivation could negatively affect performance through changes in the parasympathetic nervous system. Although sleep deprivation has been linked to a wide range of detrimental effects, few researchers have focused on the possible effects of poor sleep habits on self-control. One study found that sleep deprivation decreases self-control but increases inter-personal hostility which can lead to increased problems in the workplace (Christian and Ellis, 2011). Another study suggested that repeated nights of sleep deprivation lead to problems with self-control (Thacher, 2008). Therefore, to better understand how sleep loss affects performance and functioning, it could be useful to add a self-control approach to the Controlled Attention Model (Pilcher et al., 2013).

## Models of Self-Control

Two major models have emerged to explain why self-control can fail. One model suggests that self-control derives from limited internal resources (Muraven and Baumeister, 2000). These internal resources become exhausted when repeated acts of self-control occur, much as a muscle becomes fatigued following physical exertion. Exhausting the internal resources that allow for self-control is referred to as ego depletion. Most research on ego depletion has focused on glucose as the primary resource for these internal reserves such that when blood glucose levels are low; glucose is conserved resulting in poorer self-control (Hagger et al., 2010). Other research provides evidence for selective impairments in attentional performance due to blood glucose levels such that attentional performance was not affected by higher levels of glucose but was improved with moderate levels of glucose (Flint and Turek, 2003). However, Molden et al. (2012) found that simply rinsing the mouth with a glucose solution also increased self-control. These studies suggest a potentially complex relationship between glucose levels and glucose utilization and the human capacity for self-control. Furthermore, it is important to note that glucose may not be the only or the best indicator of the internal resources required for effective self-control. Additional research is needed to identify other potential sources that could contribute to stable levels of self-control.

A second model suggests that the loss of self-control could be the result of psychological processes and subjective motivation instead of a loss of physiological resources. Job et al. (2010) concluded that self-control is the result of a person’s belief in an internal willpower resource. Other researchers suggests that self-control could be a reflection of the human ability to switch task priorities to create an optimal balance between required tasks and desired tasks (Inzlicht et al., 2014). As such, maintaining self-control could be the result of choosing between competing goals or adapting to task priorities or even believing in an internal source of willpower. This suggests that self-control is largely an issue of correct allocation of effort (Beedie and Lane, 2012). This concept is similar to an earlier approach suggesting that performance under stress-inducing conditions could be protected by recruiting additional personal internal resources which would result in further subjective effort and physiological costs (Hockey, 1997).

One approach to more fully understanding self-control and its limitations is to integrate these two models of self-control thus combining the theory that self-control is the result of internal physiological resources with the theory that self-control can be influenced by personal choices and beliefs. Research indicates that both models can be used to explain self-control when the person is exposed to a minor stressor resulting in mild ego depletion (Vohs et al., 2012). In this situation, the person can choose a different goal or work on a different task or even choose to believe in the ability to complete the necessary work and overcome the negative effects of ego depletion. When resources are sufficiently exhausted; however, the person may not be able to execute the necessary self-control until the physiological resources are restored (Vohs et al., 2012). It seems clear that there comes a point when cognitive resources, such as motivation, cannot overcome higher levels of depletion. For example, sleep deprivation creates a strong physiological need for sleep that eventually will overwhelm the person. No matter how much the person may be motivated to stay awake (e.g., while driving a car with the potential cost of sleep onset being death), the desire and need to sleep can and does overcome any type of internal desire or decision to stay awake. Simply believing in an endless well of willpower in this case will not stop sleep onset. Sleep is only one example of a physiological necessity that can overwhelm any type of cognitive effort to resist. Many people would suggest that the human desire to eat certain types of food when the food is easily available is also difficult if not impossible to resist over time, hence the failure of many diets. Certainly, a person can only tread water for so long. In the end, no amount of cognitive desire to continue to tread water will save the person without the necessary physiological rest and restoration.

## Sleep and Self-Control

The potential interaction between poor sleep habits and self-control is not yet well understood. Sleep and sleep habits could influence our ability to monitor and successfully manage our internal resources as we make decisions and choices while awake. Part of the effect of sleep on self-control could be through processing and utilizing glucose. Glucose levels are influenced by the endogenous circadian system. The circadian system influences many daily functions such as the timing of sleep cycles, body temperature, arousal, and hormone secretion (Albrecht, 2002). Froy (2007) found that circadian rhythms also influence food processing and glucose metabolism. Moreover, the different biological components governed by the circadian system affect each other. The ability to metabolize glucose, for example, is negatively affected by sleep habits and times (Stamatakis and Punjabi, 2010) and by sleep deprivation (Leproult et al., 2003). This is one example of an endogenous loop built into the human nervous system where self-control, could be influenced by adequate levels of blood glucose and glucose utilization which, in turn, are impacted by sleep/wake cycles and circadian rhythmicity.

Sufficient sleep at night may also help restore necessary internal resources for self-control since sleep is part of an intricate physiological mechanism that restores the nervous system and contributes to long-term health and well-being. For instance, research suggests that sleep promotes brain plasticity at the level of the synapse (Ribeiro, 2012), improves immune functioning (Bryant et al., 2004), and increases well-being and longevity (Karasek, 2004). Regularly experiencing inconsistent sleep times or chronic partial sleep deprivation strains the sleep/wake regulatory processes (Barber et al., 2010). As such, sleep and self-control could create a feedback loop where good sleep habits could be an important component of the person’s capacity for self-control yet exhausted energy sources could lead to poor sleep habits through poor decisions about sleep times and the sleeping environment.

Although sleep could play a major role in self-control, relatively little research has focused on the possible link between the two. Previous research has suggested that individuals who report good sleep habits have lower psychological strain and better self-control and that sufficient sleep is necessary for replenishing self-regulatory energy (Barber et al., 2013). Similarly, Zohar et al. (2005) concluded that lack of sleep in medical residents negatively affects the necessary cognitive energy for self-control. One study; however, found that one night of sleep deprivation did not directly affect self-control as measured by aggressive responses in a game (Vohs et al., 2011). Future research is needed on a wider range of behavioral responses and decision-making processes to clarify the possible connections between sleep, sleep loss, self-control, and daily functioning. Good sleep habits is one of the key components of healthy living, and research into the complex relationships between the underlying constructs of self-control and sleep could provide a valuable foundation for understanding how humans can better function.

There is limited research examining how poor sleep habits and sleep deprivation affect elements of self-control such as subjective effort, perceived exertion, and choice. One study indicates that when allowed to make choices in which task to complete, sleep-deprived persons will select less challenging tasks than when fully rested (Engle-Friedman and Riela, 2004), signifying that sleep-deprived persons could attempt to compensate by choosing easier tasks. Similarly, Hockey et al. (1998) concluded that sleep-deprived persons will shift to less-demanding tasks to accommodate for decreased capacity. Another study found stable blood glucose levels during 48 h of sleep deprivation; however, the participants were allowed to control their speed on a walking task and voluntarily reduced their walking pace while reporting increased work load (Rodgers et al., 1995). The type of task also appears to affect the level of subjective effort but the effort expended is not always adequate to over-come the negative effects of sleep loss (Odle-Dusseau et al., 2010). Sleep loss seems to affect self-control through two mechanisms. Sleep loss negatively impacts the person’s capacity for performance as well as the person’s access to physiological energy resources (Engle-Friedman, 2014).

Despite the fact that sleep and self-control are often viewed as separate processes, recent neuroimaging studies suggest that it could be an integrated system. It is well-established that sleep deprivation is associated with decreases in brain activity, particularly in the prefrontal cortex and thalamus (e.g., Thomas et al., 2000). In addition, this decrease in brain activity has real-world implications in that sleep loss impairs prefrontal cortex functioning resulting in deficits in divergent thinking (Vartanian et al., 2014). Models of how brain activity results in self-control point to a balance between the higher-level prefrontal regions exerting self-control in a top-down fashion on the lower, subcortical regions involved in emotional responding, drives, and unconscious attitudes (Heatherton and Wagner, 2011). Furthermore, the higher level cortical structures target specific areas in the subcortical region of the brain depending on what type of behavior the individual is attempting to regulate. For example, if an individual is trying to lose weight, the areas in the subcortical region involved in hunger and satiety would be regulated in a top-down manner. In contrast, the region of the brain that is the “master” regulator, the lateral pre-frontal cortex, is consistently activated regardless of what type of behavior is being regulated (Cohen and Lieberman, 2010). Likewise, studies suggest that self-control and controlled attention activate the prefrontal cortex suggesting a common physiological mechanism (Langner and Eickhoff, 2013). It is important to note that sleep loss may potentially counter the brain’s ability to generate adequate self-control and attention. This suggests a possible mechanism for sleep to help stabilize self-control in that the decreased brain activity in sleep deprivation could impair the ability to activate the cortical structures needed for adequate self-control.

## Conclusions

To preserve self-control, it is important to balance personal effort and choices in such a way that the ability to maintain self-control remains stable while replenishing necessary resources. Unfortunately, the energy resources that allow for better self-control are more quickly depleted than replenished. As such, it is common to run low on the necessary energy reserves for self-control as each day progresses. Better understanding how sleep habits could reset and contribute to a stable set of daily energy reserves necessary for self-control opens the possibility of a new research agenda and could be one step toward overcoming the natural imbalance between available vs. used energy stores. In addition, good sleep habits could refuel a person’s ability to make more difficult choices instead of opting for the easier choice or the easier task.

Until recently, scientists in the sleep field worked separately from scientists in the cognitive based self-control field. By working to combine the two areas, we can better understand how sleep and self-control form an integrated system that provides the basis for complex decision making and capabilities seen in humans. The implications of this merger are directly relevant for many types of events in modern life. Better managing sleep and self-control capacity could improve worker performance and health. It could also help with such society issues as addictions, excessive gambling, and over-spending. Learning more about how humans manage the innate capacity for self-control could be a key in better managing daily decisions. In summary, better understanding how the interaction among good sleep habits, physiological energy reserves, and individual’s personal effort and choices impact self-control could provide a valuable means to improve long-term health and productivity.

## Conflict of Interest Statement

The authors declare that the research was conducted in the absence of any commercial or financial relationships that could be construed as a potential conflict of interest.
